# Synapses and Dendritic Spines as Pathogenic Targets in Alzheimer's Disease

**DOI:** 10.1155/2012/247150

**Published:** 2012-02-06

**Authors:** Wendou Yu, Bingwei Lu

**Affiliations:** Department of Pathology, Stanford University School of Medicine, Stanford, CA 94305, USA

## Abstract

Synapses are sites of cell-cell contacts that transmit electrical or chemical signals in the brain. Dendritic spines are protrusions on dendritic shaft where excitatory synapses are located. Synapses and dendritic spines are dynamic structures whose plasticity is thought to underlie learning and memory. No wonder neurobiologists are intensively studying mechanisms governing the structural and functional plasticity of synapses and dendritic spines in an effort to understand and eventually treat neurological disorders manifesting learning and memory deficits. One of the best-studied brain disorders that prominently feature synaptic and dendritic spine pathology is Alzheimer's disease (AD). Recent studies have revealed molecular mechanisms underlying the synapse and spine pathology in AD, including a role for mislocalized tau in the postsynaptic compartment. Synaptic and dendritic spine pathology is also observed in other neurodegenerative disease. It is possible that some common pathogenic mechanisms may underlie the synaptic and dendritic spine pathology in neurodegenerative diseases.

## 1. Introduction

The number of neurons in the human brain approximates the number of stars in the galaxy. Each of these neurons makes an average of 1000 contacts with other neurons. The result is an incredibly complex and sophisticated network made of roughly 100 trillion synapses. Communications between neurons in the brain occur primarily through synapses formed between presynaptic and postsynaptic partners. For fast synaptic transmission, there are two types of synapses: type I synapses use glutamate as the neurotransmitter and are excitatory, whereas type II synapses use gamma-amino butyric acid (GABA) as the major neurotransmitter and are inhibitory. While dendritic shafts are the main location for the inhibitory GABAergic synapses, dendritic spines, which are small membrane protrusions from dendritic shafts that contain glutamate receptors and postsynaptic density components, are the primary locations of excitatory synapses. A functional balance between neuronal excitation and inhibition is established during development for homeostatic control of neuronal excitability and is maintained into adulthood [1–4]. On the other hand, imbalances between neuronal excitation and inhibition have been associated with many neurological disorders including epilepsy [5], schizophrenia [6], fragile X syndrome [7], and autism [8].

Information can be stored in the brain by multiple synaptic mechanisms, including altered structure and chemistry of existing synapses, formation of new synapses, or elimination of old ones. Such synaptic plasticity is thought to be fundamental to learning and memory in the brain [9]. At the electrophysiological level, synaptic plasticity is reflected in processes known as long-term potentiation (LTP) and long-term depression (LTD) [10]. Excitatory synapses contain AMPA and NMDA ionotropic glutamate receptors localized on dendritic spines, with basal synaptic transmission largely mediated by the AMPA receptors. High synaptic activity opens NMDA receptors, leading to long-lasting changes in postsynaptic AMPA receptor number and LTP of synaptic transmission [11]. Alternatively, low levels of synaptic stimulation can activate NMDA receptors to produce LTD [12]. At the morphological level, LTP is generally associated with dendritic spine growth, whereas LTD can induce the removal of postsynaptic AMPA receptors and loss of spines [13–19]. It is thus not surprising that synaptic development, maintenance, and plasticity under normal physiological conditions are frequently associated with changes in the morphology and number of dendritic spines [20]. 

In many neurodegenerative diseases, particularly those exhibiting cognitive impairments such as Alzheimer's disease (AD) and Parkinson's disease (PD), dendritic spines are altered in number and shape before eventual neuronal death is observed. Changes in dendritic spine number and morphology are also found in other disease conditions such as autism, Down syndrome, drug addiction, fragile X syndrome, and schizophrenia [20–24]. It is worth emphasizing that degeneration of synapses and dendritic spines is one of the earliest features in those neurodegenerative disease conditions, prior to subsequent loss of neurons. Interventions aimed to protect the nervous system from the ravages of these disease would therefore seem more effective when the synaptic and spine pathology are prevented as early as possible. 

In this review article, we will summarize recent advances in our understanding of the molecular mechanisms underlying synaptic and dendritic spine pathology in neurodegenerative diseases, particularly in AD and PD. The readers are referred to some excellent previous reviews on the observation of synaptic and dendritic spine pathology in neurological disorders [22–25].

## 2. Synapse and Dendritic Spine Pathology in AD 

AD is the most common neurodegenerative disease and the leading cause of dementia in the elderly. Decades of intensive research have uncovered amyloid plaque and neurofibrillary tangle (NFT) as the pathological hallmarks, and soluble amyloid-*β* (A*β*) oligomers as the leading candidate for the causative agent of AD [26, 27]. However, the mechanistic link between amyloid plaque and NFT and the mechanism by which A*β* oligomer may cause cognitive impairments remains poorly defined, and there is no effective treatment for this devastating disease. Substantial evidences have accumulated indicating that the memory deficits in AD patients do not correlate well with amyloid plaque burden; instead, the loss of synaptic markers is a better predictor of clinical symptoms and disease progression [28]. Together with studies using animal AD models, these studies have lent support to the hypothesis that AD could be conceptualized as a disease of synaptic failure [28]. 

Early structural studies of postmortem tissues showed that when compared with age-matched control brains, AD brains had reduced synapse density and number of dendritic spines in the cortex and hippocampus, principal brain areas affected by the disease, and that greater loss of dendritic spines was associated with lower mental status [29, 30]. These findings suggested that progressive loss of dendritic spines is directly related to the pathogenesis of AD and represents a good indicator of disease progression. Studies of transgenic mouse models of AD have shown that, in the vicinity of amyloid plaques, there were dramatic spine loss and neurite dystrophy, structural changes that could lead to altered neuronal circuits and brain functions [31–33]. Further studies showed that the accumulation of soluble A*β* might be the culprit that leads to dendritic spines loss [34]. A*β* is the proteolytic product of a large protein called amyloid precursor protein (APP), which is cleaved by beta- and gamma-secretases to produce A*β* and other fragments of the precursor protein [35]. Interestingly, the formation and secretion of A*β* peptides are positively regulated by neuronal activity, and excess A*β* peptide can in turn depress excitatory synaptic transmission onto neurons that produce A*β* as well as nearby neurons that do not produce A*β* [36]. Thus, activity-dependent modulation of A*β* production may normally participate in a negative feedback regulatory loop to restrain neuronal hyperactivity, the impairment of which could contribute to AD pathogenesis [36]. Under normal conditions, A*β* monomers could be cleared by proteolytic enzymes like neprilysin, chaperone molecule ApoE, or the lysosomal and proteasomal pathways. However, under pathological conditions, soluble A*β* levels are increased, leading to the buildup of A*β* oligomers, which can be further sequestered into protofibrils and fibrils as seen in plaques [27]. 

Several lines of evidence support that A*β* is the primary causative agent of AD. First, genetic studies of familial forms of AD have identified rare genetic mutations that cause AD by altering the production or metabolism of A*β* peptides, leading to their aberrant accumulation [27, 37]. Soluble A*β* levels have been found to better correlate with disease progression and severity than amyloid plaques or NFTs [34]. Second, A*β* oligomers formed *in vitro* from synthetic peptides, purified from cultured cells expressing APP, or from cortex of AD patient brains can induce synaptic dysfunction and neuritic degeneration [38–41]. Third, the reduction of soluble A*β* levels using an immunization method in mouse AD models rescued the cognitive deficits [42]. However, despite the overwhelming supporting evidences, the A*β* hypothesis of AD as described above still faces challenge, since several highly publicized clinical trials targeting A*β* had failed.

## 3. Mechanisms Underlying the Synapse and Dendritic Spine Pathology in AD

The molecular mechanisms through which A*β* might cause synaptic loss and neuronal death remain uncertain. A*β* has been found to form pore-like structures with calcium channel activity, which could interfere with calcium signaling [43, 44]. A*β* can also affect LTP and LTD by modulating glutamate receptor-dependent signaling pathways [45–47] and trigger aberrant patterns of neural network activity [48]. A*β* may also cause mitochondrial dysfunction [49] and lysosomal failure [50]. 

One of the earliest clues about the mechanisms of A*β*-induced synaptic dysfunction came from studies of cultured neurons derived from Tg2576 mutant APP transgenic mice [51]. Among the synaptic changes observed were fewer and smaller postsynaptic compartments and fewer and enlarged active presynaptic compartments. Notably, the earliest observable change in synaptic components was the reduction of PSD-95, which is a master regulator of the assembly and anchoring of postsynaptic density components such as glutamate receptor subunits [52]. A*β* was shown to be the toxic agent causing these synaptic changes since the effects were blocked by gamma-secretase inhibitor treatment and recapitulated by application of synthetic A*β* to wild-type neurons [51]. Similar PSD-95-related synaptic defects were also observed in human AD brain samples [53]. The molecular mechanisms through which A*β* influences PSD-95 remain to be determined. Studies in *Drosophila* models showed that PAR-1 kinase, the fly homologue of mammalian microtubule affinity regulating kinases (MARKs), can directly phosphorylate the fly PSD-95 homologue Dlg, and this phosphorylation event caused the delocalization of Dlg from the postsynaptic membrane [54]. PAR-1/MARK kinases are known to be activated by APP or A*β* in *Drosophila* or mammalian neurons [55, 56]. It would be interesting to test whether MARKs are critical mediators of A*β* toxicity on mammalian synapses and dendritic spines.

A significant recent advance in our understanding of the mechanisms of the synaptic toxicity of A*β* has been the finding that A*β* uses LTD-related signaling mechanisms to affect synaptic function and dendritic spine morphology [45]. One of the principle mechanisms of LTD induction is the removal of AMPA receptors from the postsynaptic membrane through endocytosis. Significant parallels were found between A*β*-induced synaptic changes and LTD. Overexpression of A*β* resulted in decreased spine density and postsynaptic AMPA receptor number, through signaling molecules implicated in LTD, such as p38 MAP kinase and calcineurin. Importantly, expression of a mutant form of AMPA receptor that resists LTD-driven endocytosis blocked the morphological effects and synaptic depression induced by A*β* [45]. This study implicated the endocytosis of AMPA receptors as a major mechanism through which A*β* causes synaptic dysfunction and subsequent degeneration, but the detailed molecular mechanisms remain unclear.

Recent studies using transgenic mouse models of AD have implicated the microtubule-binding protein tau as a major mediator of the toxicity of A*β* at the postsynaptic compartment and dendritic spines. Although tau abnormality has long been observed in AD, as exemplified by the formation of NFTs by tau that accompany plaque pathology, and tau abnormality can cause neurodegeneration in the absence of plaque pathology as in frontotemporal dementia cases [57, 58], the direct involvement of tau in A*β*-induced synaptic and dendritic spine pathology may initially appear surprising, since tau is generally considered a presynaptic protein that is primarily localized to axons. In fact, the relationship between NFTs and amyloid plaques in disease pathogenesis has long been a source of considerable debate [37, 59, 60]. Studies in mice suggested that the two lesions might be causally linked. In transgenic mouse models, intracranial injection of synthetic A*β*, or crossing of APP transgenic mice with tau transgenic mice, promoted NFT pathology [61–63], and immunization of APP/Psn/tau triple transgenic mice with antibodies against A*β* reduced the levels of hyperphosphorylated tau [64]. This was consistent with earlier studies showing that the removal of tau could relieve A*β*-induced neurotoxicity in cultured neurons [65]. Together, these studies support the notion that the initiating event in AD is the accumulation of the toxic A*β* peptides, and that tau abnormality is a major downstream molecular event that contributes to disease pathogenesis [37].

How tau abnormality arises in AD is not well understood. Current efforts have focused on the role of aberrant phosphorylation of tau [27]. Previous studies have shown that A*β* could lead to abnormal activation of a number of kinases, including cyclin-dependent kinase-5 (CDK5) [66, 67], Fyn kinase [68, 69], glycogen synthase kinase-3beta (GSK3*β*) [70], and MARK [71–73], all of which promote tau hyperphosphorylation and could potentially affect synaptic structure and function. However, very few *in vivo* studies have been done to assess the roles of tau kinases or phosphatases in conferring tau toxicity and in causing AD-related memory deficit. Identification of the relevant kinases or phosphatases will provide attractive therapeutic targets for AD.

Recently studies have shown that removing endogenous tau can prevent A*β*-induced behavioral deficits in a mouse AD model expressing human APP, and block excitotoxin-induced neuronal dysfunction in both transgenic and nontransgenic mice [74]. Since current data support postsynaptic toxicity as a primary mechanism of A*β* action in causing learning and memory deficits in AD, this study raised the possibility that tau may also act in the postsynaptic compartment. Indeed, under both physiological and pathological conditions, tau was found in dendrites [75, 76], albeit the level of dendritic tau was much higher under disease conditions. Tau was known to interact with microtubules through its microtubule-binding domain to stabilize microtubule and regulate axonal transport. It has many putative phosphorylation sites and becomes hyperphosphorylated in AD patients and transgenic animal models [57, 58]. Apart from the notion that phosphorylation can lead to the dissociation of tau from the microtubules, other pathophysiological effects of this molecular event are unknown. 

A recent study has indicated that phosphorylated tau could accumulate in dendritic spines, where it may affect the synaptic trafficking and/or anchoring of glutamate receptors, thereby influencing postsynaptic function [76]. Interestingly, this effect of tau on synaptic function occurred without causing the loss of synapses or dendritic spines. This study thus revealed a critical role for tau phosphorylation in causing tau mislocalization and subsequent synaptic impairment, and it established dendritic spines as pathogenic targets of tau action. Another study provided further mechanistic insights into the dendritic function of tau [75]. Tau interacts with fyn [77], a protein tyrosine kinase that can phosphorylate tau and whose activity is increased in AD brain [78]. Ittner et al. showed that the interaction of tau with fyn leads to the targeting of fyn to dendritic spines, where fyn can phosphorylate NMDA receptor subunit 2 (GluR2), resulting in stabilization of the interaction between GluR2 and PSD-95 and enhanced excitotoxicity. Tau also shows strong interaction with PSD-95, providing further support for a dendritic role of tau besides its known axonal function. Importantly, the toxic effects of APP/A*β* were attenuated by interfering with GluR2/PSD-95 interaction with a cell-permeable peptide [75], supporting that dendritic tau-mediated fyn recruitment and GluR2/PSD-95 interaction confer A*β* toxicity at the postsynapse. 

Thus, a “tau hypothesis” has been put forward based on these recent results; A*β* triggers the phosphorylation of tau, causing tau to dissociate from the microtubules and accumulate at the dendritic compartments. Phosphorylated tau exhibits stronger interaction with Fyn and thus facilitates the targeting of fyn to dendritic spines. The targeting of fyn to postsynaptic density sensitizes the NMDA receptors and renders neurons more vulnerable to the toxicity of A*β* in the postsynaptic compartment [79]. It remains to be determined whether tau becomes hyperphosphorylated *in situ* in the dendritic spines as a result of altered kinase/phosphatase activities there, or that it becomes hyperphosphorylated elsewhere and is then transported to the dendritic spines. Nevertheless, targeting the tau-dependent pathway, for example, by reducing tau protein level, inhibiting tau kinase activities, or increasing phosphatase activities, would represent suitable new ways of treating AD. 

**Figure 1:**
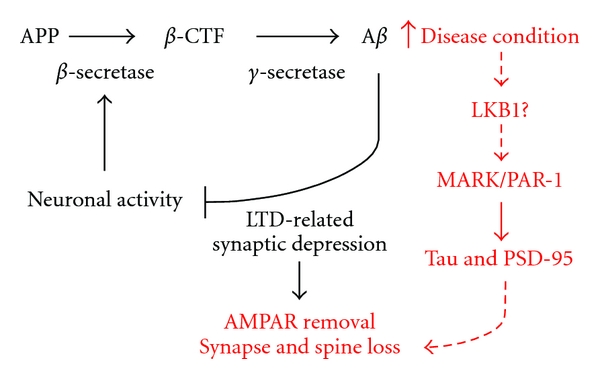
A diagram depicting the physiological and pathological roles of A*β*. The pathway in black represents the normal function of A*β* in restraining neuronal hyperactivation. In response to neuronal activation, there is upregulation of BACE, leading to overproduction of A*β*, which then acts through LTD-related mechanism involving AMPAR removal to tune down neuronal activity. In disease condition (depicted in red), however, the excessive accumulation of A*β* leads to excessive synaptic depression and AMPAR removal, which eventually results in synapse and spine loss. Based on our unpublished work (Yu et al., manuscript submitted), we propose that A*β* can act through the LKB1→MARK→tau/PSD-95 signaling cascade to cause synapse and spine loss.

In summary, we can consider the toxic effect of A*β* on neuronal synapses and dendritic spines as a normal physiological process gone awry, instead of some pathological process unique to the disease process. A*β* is continuously produced in the brain, and its production can be stimulated by neuronal activity. A*β* can then feedback on the hyperactive neuron using a LTD-related mechanism to tune down neuronal activity, for example, by promoting AMPAR removal. This process normally acts as a homeostatic mechanism to restrain neuronal hyperactivation. In the disease process, however, the buildup of A*β* tips the balance of this process toward excessive synaptic depression and AMPAR removal, resulting in synapse and spine loss (Figure 1). The molecular mechanisms involved in A*β* toxicity on synapses and dendritic spines are just beginning to be elucidated. We propose that a signaling cascade from A*β* to tau and PSD-95, involving tau kinases such as PAR-1/MARK and its activating kinase LKB1, might be involved (Figure 1).

## 4. Possible Nonneuronal Contribution to Synapse and Spine Pathology in AD

Although much of the research on the mechanisms of A*β* toxicity to synapses and spines has taken a “neuron-centric” approach, it is worth noting that other nonneuronal cell types in the brain play critical roles in the formation and maturation of synapses during development, and similar mechanisms may operate in the adult brain to mediate the effects of A*β* on neuronal synapses and dendritic spines. 

Besides providing trophic factors for neurons, glial cells have been shown to play key roles in regulating neuronal migration, axon guidance, and synapse formation [80]. In one of the better-characterized cases, astrocytes were shown to secret signals that induce synapse formation by retinal ganglion cells (RGCs). A family of extracellular matrix proteins called thrombospondins (TSPs) was identified as the synaptogenic signals coming from astrocytes [81]. The TSP receptor from the neuronal side involved in synaptogenesis was found to be the calcium channel subunit *α*2*δ*-1 [82]. Interestingly, the synapses formed by TSPs are postsynaptically silent due to the lack of surface AMPA receptors, whereas those formed by astrocyte conditioned medium are postsynaptically active, suggesting that additional factors are secreted by the astrocytes to control synaptic strength and plasticity [83]. The identity of these additional factors is currently unknown. Also, synapses are made in excess during development, and the extra synapses or weak synapses are eliminated by a process involving signals from astrocytes that induce the classical complement pathway protein C1q in neurons [84]. In addition to secreted factors, astrocytes can regulate synapse formation using contact-mediated mechanisms. Astrocytes also regulate dendritic spine morphology through a contact-mediated mechanism involving bidirectional ephrin/EphA signaling. In the hippocampus, for example, astrocytes express ephrin A3, whereas neurons express the ephrin receptor EphA4. Perturbing ephrin/EphA signaling results in defects in spine formation and maturation [85]. One can imagine that disruption of astrocyte-neuron interaction by A*β* could affect synapse and spine morphology through the above-mentioned mechanisms. In this respect, it is interesting to note that a recent study has shown that lentiviral-mediated delivery of EphB2 expression constructs in the dentate gyrus of hAPP transgenic mice reversed deficits in NMDA receptor-dependent LTP and memory impairments [86]. Whether there is glial involvement in this experimental setting has not been examined. 

The other abundant glial cells in the brain are microglia. Unlike the astrocytes, these cells are of mesodermal origin. The roles of microglia in disease pathogenesis in AD and other neurodegenerative diseases are very complex and controversial [87, 88]. This probably has to do with the diverse activities of these cells in the brain. Relevant to AD pathogenesis, microglia can promote A*β* clearance, release anti-inflammatory cytokines and neurotrophic factors on one hand, and they can also affect the activation of complement systems and elimination of synapses and spines on the other hand [88]. Thus microglia can exert neuroprotective as well as neurodegenerative effects, depending on the strength, timing, and duration of their activation. Imaging studies showed that activated microglia were found in patients with MCI [89], suggesting that neuroinflammation is an early event in the disease process. Consistent with this finding, microglial activation was observed early in a tauopathy mouse model, preceding NFT formation and roughly concurrent with synapse loss and impairment of synaptic function [90]. Interestingly, supplement of immunosuppressant FK506 to young mice attenuated tau pathology and increased lifespan, suggesting that microglia activation may contribute to disease. In another AD mouse model expressing the E693Δ mutation that causes AD by enhanced A*β* oligomerization without fibrillization, it was found that the mice displayed age-dependent accumulation of intraneuronal A*β* oligomers at around 8 months, when abnormal tau phosphorylation, and impairments of hippocampal synaptic plasticity and memory were observed. However, microglial activation was observed from 12 months, astrocyte activation from 18 months, and neuronal loss at 24 months [91]. It is not known in this case whether microglial and astrocyte activation plays a neurodegenerative role or as part of a neuroprotective, compensatory response. 

**Figure 2:**
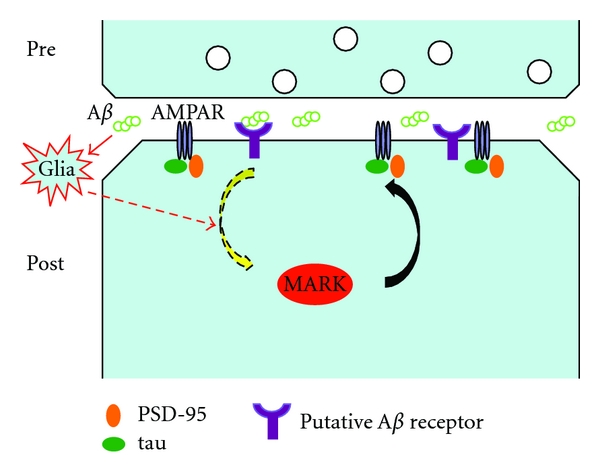
A diagram depicting a potential role of glia in mediating the synaptic toxicity of A*β*. A*β* oligomers presumably secreted from the presynaptic neuron could bind to its putative receptor on the postsynaptic cell, and this could then initiate a signaling cascade leading to activation kinases such as MARK, which then acts on tau, PSD-95, and possibly other synaptic substrates to affect AMPAR removal from the synaptic surface, leading to synapse and spine loss. Alternatively, A*β* could act on glial cells near neuronal synapses, which then release factors such as cytokines to activate signaling molecules such as MARK and cause synapse and spine loss. These two mechanisms are not mutually exclusive and could in fact occur simultaneously to mediate A*β* toxicity.

Despite the large amount of literature documenting a detrimental role for microglia and astrocyte activation in the disease process, these cells are important for neuronal heath during development and later in adult life. For example, microglia are proposed to play a surveillance role by constantly monitoring and sensing synaptic health [92], and, in addition to the critical roles, astrocytes play in synapse formation as mentioned earlier, and these cells can also control extracellular glutamate levels, remove excess extracellular K^+^, release gliotransmitters, store glucose and transform it into lactate as energy source of neurons, and scavenge ROS to protect against oxidative damages [88]. Given these essential roles of glia to neuronal function and health, it is possible that damaging of glial cells by A*β* may have equally harmful effect on the neurons eventually. In fact, there is evidence that glial cells can release ROS upon A*β* exposure [93], and glial-released cytokines may even trigger a signaling process that promotes tau hyperphosphorylation [94]. Thus, a possible role of dysfunction glial cells in AD pathogenesis should be considered, especially in the early stages of the disease process (Figure 2).

## 5. Conclusions and Future Directions

Synapse and dendritic spine pathology have been observed in the early stages of neurodegenerative diseases before neuronal death is evident, suggesting that these cellular locations represent pathogenic sites of action by the disease-causing agents early in the disease process. At least in the case of AD, there is compelling evidence supporting a pathogenic role for the synaptic and dendritic spine abnormalities. An intriguing possibility is that, as in AD, defects in the morphology and function of synapses and dendritic spines may play a critical role in the pathogenesis of PD. In fact, alterations in synaptic plasticity as represented by LTP and LTD are observed in PD, and some familial PD-associated genes have been shown to affect synapse and dendritic spine morphology and function [95, 96]. It would thus be interesting to examine whether LTP- and LTD-related signaling mechanisms are involved in PINK1/Parkin-induced synapse and dendritic spine changes. In this respect, it would also be interesting to test the potential role of dendritic tau in mediating the synaptic effects of the FPD genes. This is particularly relevant, given the identification of tau as a susceptibility factor for PD [97]. Future studies along these directions could lead to the identification of common molecular mechanisms underlying the pathogenesis of AD, PD, and possibly other neurological disorders and offer new therapeutic strategies.
